# Targeted treatment of advanced NRAS-mutated melanoma

**DOI:** 10.18632/oncotarget.21388

**Published:** 2017-09-29

**Authors:** Peter Koelblinger, Reinhard Dummer

**Affiliations:** Reinhard Dummer: Department of Dermatology, University Hospital of Zurich, Zurich, Switzerland

**Keywords:** NRAS, melanoma, binimetinib, NEMO, MEK-inhibitor

NRAS-mutations - most commonly in codon 61 (Q61R or Q61K) - can be detected in around 20% of cutaneous melanomas and have been found more frequently in nodular primary melanomas and primary tumors from skin sites with chronic UV-exposure [1]. Also, patients with NRAS-mutated locally advanced or metastatic melanoma have been proposed to undergo a more aggressive disease course with an increased frequency of CNS involvement and decreased overall survival compared to wild-type tumors [2]. Despite a suggested trend towards an improved response to immunotherapies such as programmed death 1 (PD1)-antibodies in NRAS-mutated melanoma patients [3], a substantial unmet medical need regarding targeted therapies remains in this patient subgroup. In contrast to the BRAF mutation routinely targeted clinically by effective BRAF inhibitor therapy, several attempts to directly target the mutant NRAS protein in melanoma have not even proven successful in vivo [4]. Hence, blockade of NRAS-mediated MAP-kinase pathway signaling through inhibition of the homologous kinases MEK 1 and 2 has evolved as the predominant strategy to target NRAS-mutated melanoma. Due to promising results of a phase II trial with the MEK-inhibitor binimetinib which reported a 15% obective response rate (ORR) in a cohort of 117 patients with NRAS-mutated melanoma [5], the phase III NEMO trial (NRAS melanoma and MEK inhibitor) was initiated.

Recently, the results of this randomized multicenter trial were reported in *Lancet Oncology* [6]. The trial compared binimetinib 45 mg BID with dacarbazine 1000 mg/m2 intravenously Q3W in 402 patients with NRAS-mutated locally advanced or metastatic melanoma, randomized in a 2:1 ratio (269 in the binimetinib and 133 in the dacarbazine arm). The primary endpoint of progression-free survival (PFS) could be reached with binimetinib showing superior median PFS in comparison to dacarbazine (2.8 versus 1.5 months, hazard ratio 0.62; one-sided p-value <0.001). Median overall survival (OS) did not differ significantly being 11 months in the binimetinib and 10.1 months in the dacarbazine arm. The confirmed ORR of 15.2% was significantly higher in binimetinib-treated patients (6.8% with dacarbazine) and almost identical to earlier phase II results. The median duration of response with binimetinib was 6.9 months, which is similar to what has been reported for BRAF- and MEK-inhibitor monotherapy in BRAF-mutated melanoma.

Taken together, binimetinib is the first kinase inhibitor to show clinical activity in a phase III trial in NRAS-mutated melanoma. However, despite statistical significance, the PFS improvement compared to chemotheraphy is modest, which has led to withdrawal of the new drug application for binimetinib at the FDA by the drug’s manufacturer in early 2017.

Yet, a certain subgroup of patients with NRAS-mutated melanoma may achieve increased benefit from binimetinib treatment, which is worth clinical consideration. The pre-specified subgroup of patients with prior immunotherapy treated with binimetinib in the NEMO trial (*n* = 57) showed an improved median PFS of 5.5 months. Furthermore, treatment responses in this subgroup seemed to be more durable, lasting for a median of 11 months. The response rate in immunotherapy treated patients, in turn, remained unchanged (15.8% versus 15.2%). These observations point to a certain interaction between MEK-inhibitors and immunotherapy, with a positive outcome concerning MEK-inhibitor efficacy. We have recently summarized the various effects that MEK-inhibitors can have on the interaction between tumors and the immune system and, in particular, how they can positively influence the T-cell repertoire in the tumor microenvironment [7]. Preclinical findings in this context are also supported by promising early results of a phase I clinical study with the MEK-inhibitor cobimetinib and the anti-PD-ligand 1-antibody atezolizumab in BRAF-mutant and wild-type melanoma [8].

Meanwhile, before the advent and availability of further clinical studies combining MEK-inhibitors and immunotherapy, we believe that in patients with NRAS-mutated melanoma, binimetinib represents a new and valuable treatment option after failure of immunotherapy which is worth to be considered alternative to palliative chemotherapy or merely best supportive care, regardless of its current regulatory approval status. The efficacy of binimetinib treatment may also be improved through combination with other molecules such as CDK4/6, MDM2 or ERK inhibitors in the future.

## References

[1] Lee JH (2011). Br J Dermatol.

[2] Jakob JA (2012). Cancer.

[3] Johnson DB (2015). Cancer Immunol Res.

[4] Posch C (2016). J Invest Dermatol.

[5] van Herpen CM (Sep 2014). ESMO Annual Meeting.

[6] Dummer R (2017). Lancet Oncol.

[7] Dummer R (2017). Oncoimmunology.

[8] Infante J (Nov 2016). Society for Melanoma Research Congress.

